# Gene expression profiles in primary pancreatic tumors and metastatic lesions of Ela-*c-myc *transgenic mice

**DOI:** 10.1186/1476-4598-7-11

**Published:** 2008-01-24

**Authors:** Archana Thakur, Aliccia Bollig, Jiusheng Wu, Dezhong J Liao

**Affiliations:** 1 Department of Pathology, Karmanos Cancer Institute, Wayne State University School of Medicine, 110 E. Warren Ave., Detroit, Michigan 48201, USA

## Abstract

**Background:**

Pancreatic carcinoma usually is a fatal disease with no cure, mainly due to its invasion and metastasis prior to diagnosis. We analyzed the gene expression profiles of paired primary pancreatic tumors and metastatic lesions from Ela-*c-myc *transgenic mice in order to identify genes that may be involved in the pancreatic cancer progression. Differentially expressed selected genes were verified by semi-quantitative and quantitative RT-PCR. To further evaluate the relevance of some of the selected differentially expressed genes, we investigated their expression pattern in human pancreatic cancer cell lines with high and low metastatic potentials.

**Results:**

Data indicate that genes involved in posttranscriptional regulation were a major functional category of upregulated genes in both primary pancreatic tumors (PT) and liver metastatic lesions (LM) compared to normal pancreas (NP). In particular, differential expression for splicing factors, RNA binding/pre-mRNA processing factors and spliceosome related genes were observed, indicating that RNA processing and editing related events may play critical roles in pancreatic tumor development and progression. High expression of insulin growth factor binding protein-1 (Igfbp1) and Serine proteinase inhibitor A1 (Serpina1), and low levels or absence of Wt1 gene expression were exclusive to liver metastatic lesion samples.

**Conclusion:**

We identified Igfbp1, Serpina1 and Wt1 genes that are likely to be clinically useful biomarkers for prognostic or therapeutic purposes in metastatic pancreatic cancer, particularly in pancreatic cancer where c-Myc is overexpressed.

## Background

Pancreatic cancer (PC) is the fourth leading cause of cancer death in the United States and has no cure, partly because the tumor is at advanced stage or has already metastasized at the time of diagnosis [1]. Like many other types of cancer, pancreatic cancer also shows high frequencies of overexpression and/or amplification of the c-*myc *oncogene. In one study, 43.5% of primary tumors and 31.6% of metastases showed c-Myc overexpression, in association with 32.5% and 29.4% of gene amplification in the primary and metastatic lesions, respectively [2]. c-Myc and cyclin D1 gene amplification was report 54% and 28% in 31 pancreatic cancer cell lines, respectively, indicating a high frequency of concomitant amplification of both genes [3]. Moreover, simultaneous amplification of activated k-*ras *and c-*myc *has been found in both primary tumor and lymph node metastasis, suggesting that c-Myc may collaborate with other oncogenes to promote development and progression of pancreatic cancer [4]. More direct evidence for a critical role for c-Myc in pancreatic carcinogenesis comes from Ela-c-*myc *transgenic mice that develop PC between 2–7 months of age with 100% incidence rate [5]. One-half of the pancreatic tumors that form in this mouse model are acinar cell adenocarcinomas, while the remaining half of the tumors are mixed ductal and acinar cell carcinomas embedded in dense stroma. We have recently described detailed morphological traits of the pancreatic tumors developed in this transgenic model [6,7] and, for the first time, observed spontaneous metastasis to the liver in this model. These transgenic mice are among the few animal models of liver metastasis of spontaneous PC. The whole carcinogenic process, from initiation to metastasis, is short (in only a few months time) and is initiated by only one gene.

The most devastating aspect of all types of cancer, particularly pancreatic cancer, is the emergence of metastases in organs distant from the primary tumor, and this remains the primary cause for the poor survival of patients with pancreatic cancer [8]. Therefore, a search for molecular markers that can predict poor prognosis and also serve as novel targets for the development of therapies against this most aggressive disease is warranted. Transgenic animals have been widely used to dissect the role of genes and molecular pathways in cancer [9]. Our transgenic model will help in understanding the molecular mechanisms by which metastases are generated, which is crucial for the prevention and treatment of metastatic disease. In this study we attempted to identify genes that may be responsible for the liver metastasis of pancreatic tumors in Ela-*myc *transgenic mice.

## Results

### cDNA Microarray Analysis and Global Gene Expression Profiles

**Figure 1:**
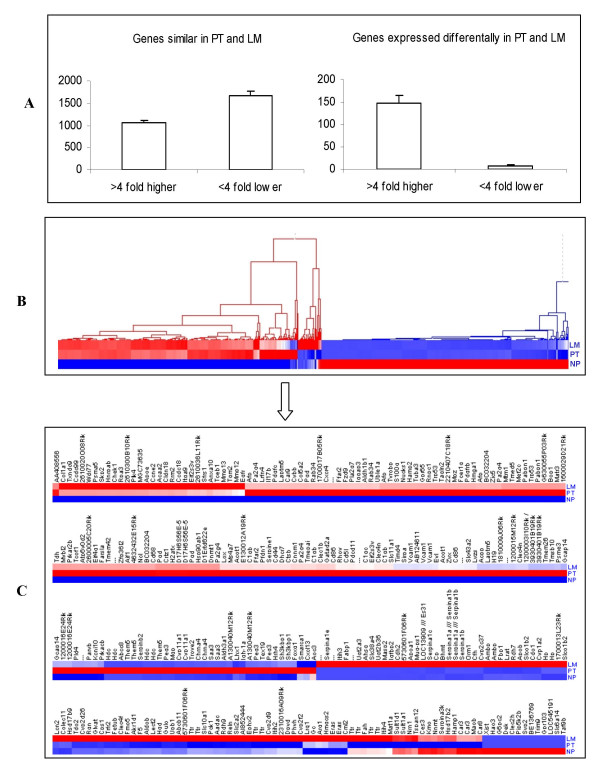
**Gene expression profiles**. **A) **Histogram showing a similar (left) and differential (right) gene expression profiles of primary pancreatic tumors and liver metastatic lesions from Ela-*c-Myc *transgenic mice compared to normal pancreas from wild type littermates. **B) **Hierarchical clustering of differentially expressed genes. Clustering tree illustrate the expression pattern and similarity in primary pancreatic tumors (labeled as PT) and liver metastatic lesions (labeled as LM) compared to normal pancreas (labeled as NP) indicated by color bars. **C) **Shows only the differentially expressed gene profile with at least a four-fold change (≤4 or ≥4) indicated by color bars. (blue-down regulated and red up-regulated).

Microarray signal values were calculated from the multiple probes present on each chip for each condition and each condition was repeated at least three times. The relative intensity (fold change) of gene expression levels in the primary tumors (PT) compared to the normal pancreas (NP) is shown in Figure 1A (left panel) and fold change in gene expression in liver metastatic (LM) lesions compared to PT are presented in Figures 1A (right panel).

Cluster analysis was used to display the gene expression data of those, which showed 4-fold higher or 4-fold lower expression levels in PT and LM compared to NP samples. Before clustering, a filtering procedure eliminated genes with uniformly low expression or with low expression variation across the replicates. A large number of genes in PT and LM showed different expression from NP. However, the majority of genes did not show obvious distinction in their expression pattern between the PT and LM (Fig. 1B), except for a small number of genes (boxed area in Fig. 1B expanded in Fig. 1C), suggesting that the LM largely retain the properties of the primary tumors.

### Identification of potential tumor promoting genes in c-myc-induced pancreatic tumors

Expressed genes were categorized on the basis of their functional properties, which showed at least 4-fold higher, or 4-fold lower expression levels in primary or metastatic pancreatic tumors compared to normal pancreas. Table 1 shows genes whose expression was upregulated in PT compared to NP (relative fold change) and also shows the relative fold change in LM compared to PT samples. Many upregulated genes such as Birc5, Ccna2, Ccnb1, Ccnb2, Mcm7, Nap1l1, Rad51, Smc4l1, Smc2l1, Rsk4, sfrs1, and sfrs2 (please see Table 1 for their full names) showed 5–20 fold higher expression levels, very few showed exceptionally high fold changes, for example calcium binding protein-S100g showed 109 fold higher expression level in PT than in NP. A large number of upregulated genes in PT belonged to the functional categories known for cell proliferation and cell cycle regulation, chromosomal organization and biogenesis, and RNA processing and modification. In Table 2, we show the genes whose expression was down regulated in PT compared to NP samples (relative fold change) as well as the fold change in LM compared to PT samples. Down regulation of some of the genes in Table 2 including Col4a4, Pcdh17, Muc2, Muc13 (please see Table 2 for their full names) has been shown to modulate cell adhesion and apoptosis.

**Table 1:** Upregulated genes in primary pancreatic tumors. Relative fold change in primary pancreatic tumors compared to normal pancreas (PT/NP) and in liver metastatic lesions compared to primary pancreatic tumors (LM/PT).

**Entrez Gene**	Fold change LM*/PT*	Fold change PT/NP*	**Gene Symbol**	**Gene description**	Ref.*
**Mitochondrial ribosomal subunits**					
**77721**	1.0	4.2	Mrps5	Mitochondrial ribosomal protein S5	
**69527**	1.0	4.5	Mrps9	Mitochondrial ribosomal protein S9	
**94063**	1.0	4.1	Mrpl16	Mitochondrial ribosomal protein L16	
**56284**	0.9	5.0	Mrpl19	Mitochondrial ribosomal protein L19	
**66407**	0.8	4.1	Mrps15	Mitochondrial ribosomal protein S15	
**64655**	1.2	7.6	Mrps22	Mitochondrial ribosomal protein S22	
**64658**	1.0	4.1	Mrps25	Mitochondrial ribosomal protein S25	
**Nucleolar and nucleosome assembly proteins**					
**53605**	0.9	13.5	Nap1l1	Nucleosome assembly protein 1-like 1	**10, 11**
**110109**	0.9	4.3	Nol1	Nucleolar protein 1	
**52530**	1.0	10.0	Nola2	Nucleolar protein family A, member 2	
**100608**	1.1	9.4	Noc4l	Nucleolar complex associated 4 homolog	
**55989**	0.8	6.3	Nol5	Nucleolar protein 5	
**67134**	0.9	7.8	Nol5a	Nucleolar protein 5A	
**Small nuclear ribonucleoprotein complex**					
**68981**	1.1	8.7	Snrpa1	Small nuclear ribonucleoprotein polypeptide A'	
**20638**	0.9	8.3	Snrpb	Small nuclear ribonucleoprotein B	
**20641**	1.1	7.1	Snrpd1	Small nuclear ribonucleoprotein D1	
**67332**	1.1	7.4	Snrpd3	Small nuclear ribonucleoprotein D3	
**69878**	1.1	6.9	Snrpf	Small nuclear ribonucleoprotein polypeptide F	
**666609**	1.0	7.6	Snrpg	small nuclear ribonucleoprotein polypeptide G	
**Splicing factor**					
**110809**	1.1	5.5	Sfrs1	Splicing factor, arginine/serine-rich 1 (ASF/SF2)	
**20382**	1.1	5.1	Sfrs2	Splicing factor, arginine/serine-rich 2 (SC-35)	
**20383**	1.1	5.0	Sfrs3	Splicing factor, arginine/serine-rich 3 (SRp20)	
**81898**	1.2	5.2	Sf3b1	Splicing factor 3b, subunit 1	**15**
**66125**	1.2	8.0	Sf3b5	Splicing factor 3b, subunit 5	**15**
**225027**	1.2	4.1	Sfrs7	Splicing factor, arginine/serine-rich 7	
**RNA binding and pre-mRNA processing factors**					
**28000**	1.1	4.7	Prpf19	PRP19/PSO4 pre-mRNA processing factor 19 homolog	
**68988**	1.1	5.0	Prpf31	PRP31 pre-mRNA processing factor 31 homolog (yeast)	
**56194**	1.1	5.8	Prpf40a	PRP40 pre-mRNA processing factor 40 homolog A (yeast)	
**56275**	0.9	5.5	Rbm14	RNA binding motif protein 14	
**67071**	1.0	16.2	Rps6ka6 (Rsk4)	Ribosomal protein S6 kinase polypeptide 6	
**Spliceosome complex**					
**81898**	1.2	5.2	Sf3b1	Splicing factor 3b, subunit 1	**15**
**66125**	1.2	8.0	Sf3b5	Splicing factor 3b, subunit 5	**15**
**20382**	1.1	4.9	Sfrs2	Splicing factor, arginine/serine-rich 2 (SC-35)	
**68981**	1.1	8.7	Snrpa1	Small nuclear ribonucleoprotein polypeptide A'	
**20638**	0.9	8.3	Snrpb	Small nuclear ribonucleoprotein B	
**20641**	1.1	7.1	Snrpd1	Small nuclear ribonucleoprotein D1	
**69878**	1.1	6.9	Snrpf	Small nuclear ribonucleoprotein polypeptide F	
**666609**	1.0	7.6	Snrpg	small nuclear ribonucleoprotein polypeptide G	
**Cell proliferation and cell cycle regulation related genes**					
**12428**	1.0	16.6	Ccna2	Cyclin A2	
**268697**	1.2	11.2	Ccnb1	Cyclin B1	
**12429**	1.1	17.9	Ccnb1-rs1	Cyclin B1, related sequence 1	
**12442**	0.9	17.8	Ccnb2	Cyclin B2	**15, 25**
**12448**	1.3	4.9	Ccne2	Cyclin E2	
**12449**	0.9	8.9	Ccnf	Cyclin F	
**17216**	0.9	9.0	Mcm2	Minichromosome maintenance deficient 2	**14**
**17215**	0.9	8.6	Mcm3	Minichromosome maintenance deficient 3	
**17217**	1.2	8.6	Mcm4	Minichromosome maintenance deficient 4	**10**
**17218**	1.0	11.8	Mcm5	Minichromosome maintenance deficient 5	
**17219**	1.1	20.1	Mcm6	Minichromosome maintenance deficient 6	
**17220**	0.9	11.0	Mcm7	Minichromosome maintenance deficient 7	**14**
**70024**	1.1	6.3	Mcm10	Minichromosome maintenance deficient 10	
**11799**	1.0	11.1	Birc5	Baculoviral IAP repeat-containing 5	
**12211**	1.0	4.4	Birc6	Baculoviral IAP repeat-containing 6	
**12189**	1.0	5.5	Brca1	Breast cancer 1	
**70099**	0.9	17.3	Smc4l1	Structural maintenance of chromosomes 4	
**19361**	1.0	15.1	Rad51	RAD51 homolog (S. cerevisiae)	
**Cell adhesion and migration**					
**12774**	1.1	6.7	Ccr5	Chemokine (C-C motif) receptor 5	
**56492**	1.4	6.6	Cldn18	Claudin 18	25
**Cell communication and signal trasduction**					
**75590**	0.8	30.3	Dusp9	Dual specificity phosphatase 9	
**67071**	1.0	16.2	Rps6ka6 (Rsk4)	Ribosomal protein S6 kinase polypeptide 6	
**12774**	1.1	6.7	Ccr5	Chemokine (C-C motif) receptor 5	
**56275**	0.9	5.5	Rbm14	RNA binding motif protein 14	
**12309**	0.7	109.4	S100g	S100 calcium binding protein G	**10, 25**
**Apoptosis regulation related**					
**11799**	1.0	11.1	Birc5	Baculoviral IAP repeat-containing 5	**16**
**17218**	1.0	11.8	Mcm5	Minichromosome maintenance deficient 5,	
**17319**	1.1	6.8	Mif	Macrophage migration inhibitory factor	
**Chromosome organization and biogenesis**					
**14211**	1.1	12.7	Smc2l1	Structural maintenance of chromosomes 2	
**70099**	0.9	17.3	Smc4l1	Structural maintenance of chromosomes 4	
**226026**	1.0	5.4	Smc5l1	Structural maintenance of chromosomes 5	
**19361**	1.0	15.1	Rad51	RAD51 homolog (S. cerevisiae)	**12**
**12189**	1.0	5.5	Brca1	Breast cancer 1	
**53605**	0.9	13.5	Nap1l1	Nucleosome assembly protein 1-like 1	**10, 11**
**17216**	0.9	9.0	Mcm2	Minichromosome maintenance deficient 2 mitotin	
**17218**	1.0	11.8	Mcm5	Minichromosome maintenance deficient 5	
**Transcriptional regulator**					
**22431**	0.6	2.7	Wt1	Wilms' tumor suppressor gene	**57**

**NP = Normal pancreas; PT = Primary pancreatic tumor; LM = liver metastatic lesion; Ref**.* = References identifying genes previously shown to have deregulated expression in pancreatic cancer

**Table 2:** Downregulated genes in primary pancreatic tumors. Relative fold change in primary pancreatic tumors compared to normal pancreas (PT/NP) and in liver metastatic lesions compared to primary pancreatic tumors (LM/PT)

**Entrez Gene#**	**Fold change LM/PT**	**Fold change PT/NP**	**Gene Symbol**	**Gene description**	Ref.*
**Cell adhesion, motility and migration**					
**12340**	0.84	-11.6	Capza1	Capping protein (actin filament) muscle Z-line, alpha 1	**16**
**12829**	0.98	-10.8	Col4a4	Procollagen, type IV, alpha 4	
**13643**	1.02	-7.6	Efnb3	Ephrin B3	
**215384**	1.03	-8	Fcgbp	Fc fragment of IgG binding protein	
**16855**	1.00	-6.4	Lgals4	Lectin, galactose binding, soluble 4	
**17831**	1.02	-40	Muc2	Mucin 2	
**219228**	1.51	-18.8	Pcdh17	Protocadherin 17	
**68799**	1.20	-7.2	Rgmb	RGM domain family, member B	
**16855**	1.00	-6.4	Lgals4	Lectin, galactose binding, soluble 4	
**Cell communication and signal trasduction**					
**12154**	1.09	-4	Bmp10	Bone morphogenetic protein 10	
**13643**	1.02	-7.6	Efnb3	Ephrin B3	
**14463**	1.01	-8	Gata4	GATA binding protein 4	
**15874**	0.96	-40	Iapp	Islet amyloid polypeptide	
**16333**	0.85	-23.2	Ins1	Insulin I	
**14526**	0.91	-21.6	Gcg	Glucagon	
**70497**	0.86	-8	Arhgap17	Rho GTPase activating protein 17	
**232201**	0.83	-7.6	Arhgap25	Rho GTPase activating protein 25	
**110052**	1.00	-8.4	Dek	DEK oncogene (DNA binding)	**16**
**14915**	0.98	-13.6	Guca2a	Guanylate cyclase activator 2a (guanylin)	
**212307**	0.81	-7.2	Mapre2	Microtubule-associated protein, RP/EB family, member 2	
**20844**	1.15	-13.6	Stam	Signal transducing adaptor molecule	
**66042**	0.85	-14.8	Sostdc1	Sclerostin domain containing 1	
**68799**	1.20	-7.2	Rgmb	RGM domain family, member B	
**80718**	0.91	-6.4	Rab27b	RAB27b, member RAS oncogene family	
**18386**	0.93	-6	Oprd1	Opioid receptor, delta 1	
**67709**	0.88	-13.6	Reg4	Regenerating islet-derived family, member 4	
**Cell cycle and cell proliferation**					
**76499**	1.02	-8.8	Clasp2	CLIP associating protein 2	
**16333**	0.85	-23.2	Ins1	Insulin I	
**16334**	0.98	-40	Ins2	Insulin II	
**212307**	0.81	-7.2	Mapre2	Microtubule-associated protein, RP/EB family, member 2	
**22268**	0.90	-6	Upk1b	Uroplakin 1B	
**14526**	0.91	-21.6	Gcg	Glucagon	
**212307**	0.81	-7.2	Mapre2	Microtubule-associated protein, RP/EB family, member 2	
**57263**	1.11	-28	Retnlb	Resistin like beta	
**12154**	1.09	-4	Bmp10	Bone morphogenetic protein 10	
**17831**	1.02	-40	Muc2	Mucin 2	**19**
**17063**	0.91	-60	**Muc13**	Mucin 13, epithelial transmembrane	
**Transporter and binding activity**					
**11773**	1.09	-14.8	Ap2m1	Adaptor protein complex AP-2, mu1	
**80718**	0.91	-6.4	Rab27b	RAB27b, member RAS oncogene family	
**56185**	1.00	-19.2	Hao3	Hydroxyacid oxidase (glycolate oxidase) 3	
**110052**	1.00	-8.4	Dek	DEK oncogene (DNA binding)	**16**
**12829**	0.98	-10.8	Col4a4	Procollagen, type IV, alpha 4	
**16467**	1.13	-11.6	Atcay	Ataxia, cerebellar, Cayman type homolog (human)	
**13487**	0.95	-20	Slc26a3	Solute carrier family 26, member 3	
**216156**	0.92	-4	Wdr18	WD repeat domain 18	
**69008**	1.23	-6.4	Cab39l	Calcium binding protein 39-like	
**12351**	0.84	-4	Car4	Carbonic anhydrase 4	
**72832**	0.93	-14.8	Crtac1	Cartilage acidic protein 1	
**75600**	1.20	-8	Calml4	Calmodulin-like 4	
**Apoptosis**					
**15874**	0.96	-40	Iapp	Islet amyloid polypeptide	
**17831**	1.02	-40	Muc2	Mucin 2	**19**
**71361**	1.15	-8	Amid	Apoptosis-inducing factor, mitochondrion-associated 2	
**16334**	0.98	-40	Ins2	Insulin II	
**17063**	0.91	-60	**Muc13**	Mucin 13, epithelial transmembrane	
**Transcription activity**					
**109275**	0.94	-4	Actr5	ARP5 actin-related protein 5 homolog (yeast)	
**71458**	0.89	-6	Bcor	Bcl6 interacting corepressor	
**14463**	1.01		Gata4	GATA binding protein 4	
**Epigenetic and chromatin modification**					
**213742**	1.00	-8.8	Xist	Inactive X specific transcripts	
**75796**	0.86	-4	Cdyl2	Chromodomain protein, Y chromosome-like 2	
**Inflammatory and immune response**					
**21786**	0.90	-10.8	Tff3	Trefoil factor 3, intestinal	
**15101**	0.90	-7.6	H60	Histocompatibility 60	
**94071**	1.00	-4	Clec2h	C-type lectin domain family 2, member h	
**Cell differentiation**					
**12154**	1.09	-4	Bmp10	Bone morphogenetic protein 10	
**14463**	1.01	-8	Gata4	GATA binding protein 4	
**72324**	0.86	-4	Plxdc1	Plexin domain containing 1	
**20755**	1.31	-16	Sprr2a	Small proline-rich protein 2A	
**22268**	0.90	-6	Upk1b	Uroplakin 1B	
**75770**	0.85	-8.4	Brsk2	BR serine/threonine kinase 2	
**Maintenance of cell polarity and shape**					
**76499**	1.02	-8.8	Clasp2	CLIP associating protein 2	
**20755**	1.31	-16	Sprr2a	Small proline-rich protein 2A	

**Ref**.* = References identifying genes previously shown to have deregulated expression in pancreatic cancer

**Figure 2:**
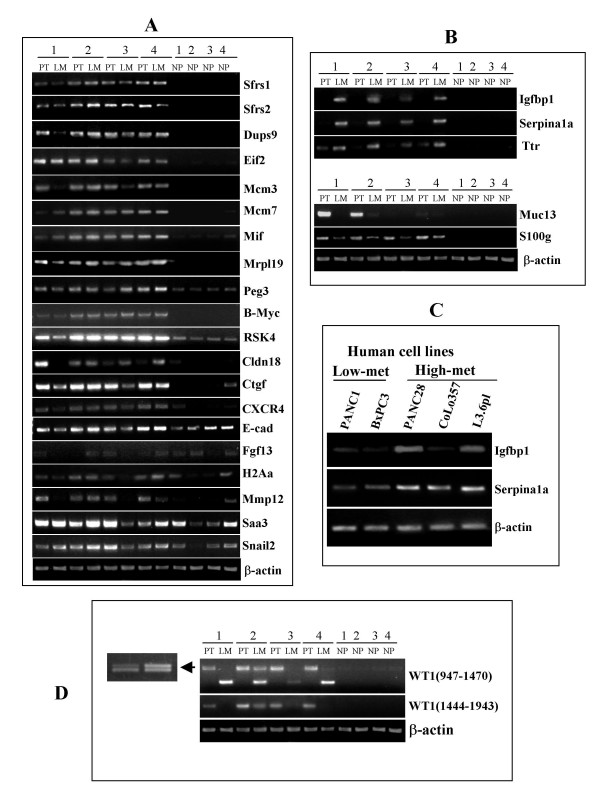
**Selected genes showing up- or down regulation of mRNA expression by semi quantitative RT-PCR**. **A) **All selected genes showed expression pattern similar to microarray data upon confirmation by sqRT-PCR. A representative data from four Ela-*c-myc *pancreatic tumors, liver metastatic lesions and normal pancreas is presented. **B) **RT-PCR showing representative differentially expressed genes in liver metastatic lesions compared to primary pancreatic tumors and normal pancreas. **C) **Two genes, Igfbp1 and Serpina1a, were verified in human pancreatic cancer cell lines with high (High-met) and low metastatic (Low-met) potentials. Expression patterns of both genes were consistent with the murine microarray and RT-PCR data. **D) **RT-PCR was performed on RNA from primary pancreatic tumors (PT), liver metastatic lesions (LM) and normal pancreas (NP) with three overlapping primer sets spanning the region from exon 1 to 10. Primary pancreatic tumors showed presence of both wild type Wt1 and Wt1 variant without exon 5, while metastatic lesions either lacked expression or had low levels of Wt1 gene expression (showed a smaller size non-specific PCR product only).

Selected genes (highlighted in Table 1, 2 and 3) from various functional categories were further verified by RT-PCR for their expression patterns (Fig. 2A). This selection was based on results in the literature indicating a direct or indirect role for each candidate gene in RNA processing, cell signaling, cell proliferation or apoptosis and cell adhesion and motility activities resulting in tumor growth and tumor progression. Many of these genes listed in Table 1and 2, such as Birc5, Brca1, Ccnb2, CXCR4, Mcm2, Mcm4, Mcm7, Nap1l1, Rad51, Sf3b, S100g [10-17]have been shown to be upregulated, while Cldn18, Muc2, Muc13, and b-myc [18-21]are shown to be down regulated in human pancreatic cancer as well as other types of cancer (please see Table 1 and 2for their full names). However, strong expression of Muc13 in 50% of samples as well as b-*myc *in pancreatic cancer cells was unexpected and needs further characterization.

**Table 3:** Upregulated genes in liver metastatic lesions. Relative fold change in liver metastatic lesions compared to primary pancreatic tumors (LM/PT) and in primary pancreatic tumors compared to normal pancreas (PT/NP)

**Entrez Gene #**	**Fold change LM/PT**	**Fold change PT/NP**	**Gene Symbol**	**Gene description**	Ref.*
**Transporter activity**					
**27413**	5.1	0.6	Abcb11	ATP-binding cassette, sub-family B (MDR/TAP), member 11	
**12870**	11.8	0.9	Cp	Ceruloplasmin	
**107141**	4.2	1.1	Cyp2c37	Cytochrome P450, family 2. subfamily c, polypeptide 37	
**76279**	9.1	0.6	Cyp2d26	Cytochrome P450, family 2. subfamily d, polypeptide 26	
**13107**	7.8	0.3	Cyp2f2	Cytochrome P450, family 2, subfamily f, polypeptide 2	
**14263**	11.3	0.4	Fmo5	Flavin containing monooxygenase 5	
**268756**	9.0	0.5	Gulo	Gulonolactone (L-) oxidase	
**20493**	8.3	0.3	Slc10a1	Solute carrier family 10 member 1	
**69354**	8.3	1.0	Slc38a4	Solute carrier family 38, member 4	
**28253**	4.9	0.9	Slco1b2	Solute carrier organic anion transporter family, member 1b2	
**Cellular metabolism**					
**67758**	10.6	0.3	Aadac	Arylacetamide deacetylase (esterase)	
**208665**	11.4	0.3	Akr1d1	Aldo-keto reductase family 1, member D1	
**11806**	43.3	0.8	Apoa1	Apolipoprotein A-I	
**238055**	12.2	0.6	Apob	Apolipoprotein B	
**12116**	33.0	0.6	Bhmt	Betaine-homocysteine methyltransferase	
**14121**	9.3	0.7	Fbp1	Fructose bisphosphatase 1	
**227231**	33.6	0.3	Cps1	Carbamoyl-phosphate synthetase 1	
**231396**	14.8	1.0	Ugt2b36	UDP glucuronosyltransferase 2 family, polypeptide B36	
**15233**	6.9	0.4	Hgd	Homogentisate 1, 2-dioxygenase	
**15483**	4.2	0.2	Hsd11b1	Hydroxysteroid 11-beta dehydrogenase 1	
**13850**	7.8	0.4	Ephx2	Epoxide hydrolase 2, cytoplasmic	
**13077**	7.0	0.8	Cyp1a2	Cytochrome P450, family 1, subfamily a, polypeptide 2	
**54150**	18.2	0.5	Rdh7	Retinol dehydrogenase 7	
**72094**	7.4	1.0	Ugt2a3	UDP glucuronosyltransferase 2 family, polypeptide A3	
**103149**	6.3	0.6	Upb1	Ureidopropionase, beta	
**16922**	5.4	0.4	Phyh	Phytanoyl-CoA hydroxylase	
**Calcium binding activity**					
**19733**	11.6	0.5	Rgn	Regucalcin	
**14067**	6.9	0.5	F5	Coagulation factor V	
**16426**	48.0	1.0	Itih3	Inter-alpha trypsin inhibitor, heavy chain 3	
**Cell organization and biogenesis**					
**11625**	40.5	0.9	Ahsg	Alpha-2-HS-glycoprotein	
**19699**	5.5	0.5	Reln	Reelin	
**16008**	6.0	1.0	Igfbp2	Insulin-like growth factor binding protein 2	
**14080**	74.7	1.0	Fabp1	Fatty acid binding protein 1, liver	
**Protease Inhibitor activity**					
**20700**	24.9	4.1	**Serpina1a**	Serine (or cysteine) peptidase inhibitor, clade A, member 1a	**25, 51**
**20702**	100.1	0.4	Serpina1c	Serine (or cysteine) peptidase inhibitor, clade A, member 1c	
**59083**	22.8	0.3	Fetub	Fetuin beta	
**Inflammatory and Immune response**					
**12628**	4.4	1.1	Cfh	Complement component factor h	
**17175**	4.5	1.0	Masp2	Mannan-binding lectin serine peptidase 2	
**11625**	40.5	0.9	Ahsg	Alpha-2-HS-glycoprotein	
**15439**	14.4	7.6	Hp	Haptoglobin	
**18405**	15.8	1.4	Orm1	Orosomucoid 1	
**12583**	8.4	0.8	Cdo1	Cysteine dioxygenase 1, cytosolic	
**13850**	7.8	0.4	Ephx2	Epoxide hydrolase 2, cytoplasmic	
**11699**	90.2	0.2	Ambp	Alpha 1 microglobulin/bikunin	**28**
**Cell Adhesion**					
**12558**	4.7	1.0	Cdh2	Cadherin 2	
**14067**	6.9	0.5	F5	Coagulation factor V	
**16008**	6.0	1.0	Igfbp2	Insulin-like growth factor binding protein 2	
**19699**	5.5	0.5	Reln	Reelin	
**17175**	4.5	1.0	Masp2	Mannan-binding lectin serine peptidase 2	
**14080**	74.7	1.0	Fabp1	Fatty acid binding protein 1, liver	
**Cell growth and cell cycle**					
**14080**	74.7	1.0	Fabp1	Fatty acid binding protein 1, liver	
**16008**	6.0	1.0	Igfbp2	Insulin-like growth factor binding protein 2	
**11625**	40.5	0.9	Ahsg	Alpha-2-HS-glycoprotein	
**Cell motility and migration**					
**12558**	4.7	1.0	Cdh2	Cadherin 2	
**19699**	5.5	0.5	Reln	Reelin	
**16841**	4.8	0.6	Lect2	Leukocyte cell-derived chemotaxin 2	
**20315**	4.5	0.1	Cxcl12	Chemokine (C-X-C motif) ligand 12	
**12738**	2.8	0.3	Cldn2	Claudin 2	
**Cell communication and Signal Transduction**					
**208665**	11.4	0.3	Akr1d1	Aldo-keto reductase family 1, member D1	
**22139**	38.8	0.1	**Ttr**	Transthyretin	
**16008**	6.0	1.0	Igfbp2	Insulin-like growth factor binding protein 2	
**20526**	13.1	0.3	Slc2a2	Solute carrier family 2, member 2	
**238055**	12.2	0.6	Apob	Apolipoprotein B	
**50765**	4.4	0.7	Trfr2	Transferrin receptor 2	
**107146**	4.7	0.7	Glyat	Glycine-N-acyltransferase	
**51811**	5.7	0.7	Clec4f	C-type lectin domain family 4, member f	
**14080**	74.7	1.0	Fabp1	Fatty acid binding protein 1, liver	
**56720**	4.0	0.8	Tdo2	Tryptophan 2,3-dioxygenase	
**11625**	40.5	0.9	Ahsg	Alpha-2-HS-glycoprotein	
**353283**	4.1	42.0	Eras	ES cell-expressed Ras	
**19699**	5.5	0.5	Reln	Reelin	
**16006**	28.1	0.7	I**gfbp1**	Insulin-like growth factor binding protein 1	**28,30,31**

**Ref**.* = References identifying genes previously shown to have deregulated expression in pancreatic cancer

**Figure 3:**
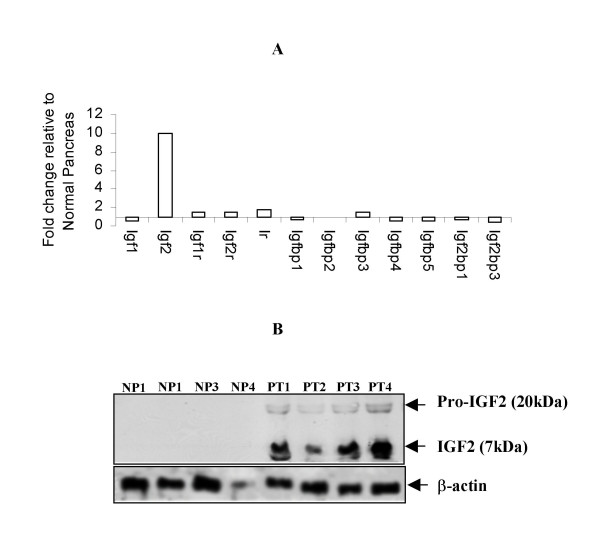
**Expression of IGF family genes and proteins**. **A) **Microarray data show that expression of Igf2 is about 10 fold higher in pancreatic tumors compared to liver metastatic lesions and normal pancreas from Ela-*myc *transgenic mice. While other IGF family proteins only showed modest change. **B) **Western blot analysis of Insulin like growth factors and their receptor proteins. Western blot was performed in cell lysates prepared from primary pancreatic tumors (PT), liver metastatic lesions (LM) from Ela-*c-myc *transgenic mice and normal pancreas (NP) from wild type littermates. Consistent with microarray data, PT samples showed noticeably higher protein levels compared to NP samples. A representative data from four PT and four NP samples are presented.

We evidenced notable changes in the family members of insulin-like growth factor (Igf). While Igf1 expression was slightly decreased in tumors compared with normal pancreas in the wild type littermates, Igf2 expression was dramatically increased (Fig 3A). All three receptors for Igf1 and Igf2 showed only slight increase in their expression, on the other hand all Igf binding proteins (Igfbp1, Igfbp2, Igfbp3, Igf2bp1 etc.) were downregulated compared to normal pancreas. Western blot analysis confirmed increased expression of cleaved, active form of Igf2 (Fig 3B).

### Identification of potential metastasis promoting genes in c-*myc *induced pancreatic tumors

As mentioned above, we identified a small number of genes that were under various functional categories in metastatic tissues, which were either significantly upregulated or downregulated compared to PT. Interestingly, genes that were downregulated in liver metastatic lesions were comparatively much fewer than upregulated genes. Table 3 shows 4-fold higher and Table 4, 4-fold lower expression levels in LM compared to PT. Most of the highly upregulated genes such as Cp, Apoa1, Ttr in liver metastatic lesions are known biomarkers for the detection of ovarian or other types of cancer [22-24]. Other highly upregulated genes were related to protease inhibition such as Serpina1a, Serpina1c, Ambp [25-27]and insulin growth factor binding proteins such as Igfbp1 and Ifgbp2 [28-31], which have been shown to be upregulated in human pancreatic cancer as well as in the animal models of either pancreatic cancer or other types of cancer. For the verification of some of these genes, we selected two upregulated and two downregulated genes, that showed striking differences from primary pancreatic tumors. In line with our mocroarray data, all LM samples verified by RT-PCR showed highly consistent results (Figure 2B).

**Table 4:** Downregulated genes in liver metastatic lesions. Relative fold change in liver metastatic lesions compared to primary pancreatic tumors (LM/PT) and in primary pancreatic tumors compared to normal pancreas (PT/NP)

**Entrez Gene #**	**LM/PT**	**PT/NP**	**Gene Symbol**	**Gene description**	Ref.*
**Cell communication and Signal transduction**					
**22329**	0.5	23.5	Vcam1	Vascular cell adhesion molecule 1	
**58194**	0.4	4.0	Sh3kbp1	SH3-domain kinase binding protein 1	
**15186**	0.1	15.0	Hdc	Histidine decarboxylase	
**11438**	0.2	4.9	Chrna4	Cholinergic receptor, nicotinic, alpha polypeptide 4	
**12524**	0.6	4.6	Cd86	CD86 antigen	
**93761**	0.2	4.2	Smarca1	SWI/SNF related, regulator of chromatin, subfamily a, member 1	
**Cell motility and migration**					
**12767**	0.7	4.7	**Cxcr4**	Chemokine (C-X-C motif) receptor 4	**25**
**17381**	2.8	7.6	**Mmp12**	Matrix metallopeptidase 12	**16**
**11438**	0.2	4.9	Chrna4	Cholinergic receptor, nicotinic, alpha polypeptide 4	
**Cell Adhesion**					
**12505**	0.6	5.3	Cd44	CD44 antigen	**11**
**22329**	0.5	23.5	Vcam1	Vascular cell adhesion molecule 1	
**Cell death and apoptosis**					
**18616**	0.2	11.2	**Peg3**	Paternally expressed 3	
**11801**	0.6	31.1	Cd5l	CD5 antigen-like	
**58194**	0.4	4.0	Sh3kbp1	SH3-domain kinase binding protein 1	
**Inflammatory and Immune response**					
**20210**	0.1	14.1	**Saa3**	Serum amyloid A 3	
**58194**	0.4	4.0	Sh3kbp1	SH3-domain kinase binding protein 1	
**15186**	0.1	15.0	Hdc	Histidine decarboxylase	

**Ref**.* = References identifying genes previously shown to have deregulated expression in pancreatic cancer

### Decreased or lost expression of Wt1 mRNA in primary pancreatic tumors

Wt1 is a transcription factor and has been found to be overexpressed in several types of cancers with poor prognosis. Our microarray data showed two-fold higher expression of the Wt1 gene in PT samples compared to NP samples. RT-PCR with a pair of primers that amplify exons 1 to 7 could detect Wt1 mRNA in PT but not in NP and LM (Fig. 2D). Interestingly, liver metastatic lesions expressed a lower molecular species of mRNA. We purified the higher band from primary tumors and the lower band from liver metastatic lesions and sequenced the PCR products. The results showed that the Wt1 mRNA in PT contained both wild type Wt1 and Wt1 variant without exon 5 (-51 nt). The slight difference in length could be visualized on agarose gel when the PCR products were separated further (Fig. 2D, amplified zone). On the other hand, sequencing results of the band in liver metastatic lesions showed that it was a product of Uroc1 (urocanase domain containing 1) gene, not Wt1. Comparison of the primer sequences with the mouse Uroc1 cDNA (NM_144940) showed high homology, and therefore a non-specific band (Uroc1) was amplified with this primer pair. Since human Uroc1 gene is highly expression in hepatoblastoma than in fetal liver [32], it is possible that Uroc1 is preferentially expressed in liver tumors and thus may serve as a marker. PCR with another pair of primers that amplified nt1444-1943 region of the mRNA also showed that LM expressed much lower levels of Wt1. Considering that a tissue is heterogeneous in cell types, it is reasonable to assume that the Wt1 mRNA detected in LM was derived from stromal tissue whereas the cancer cells might have lost Wt1 expression.

### Real-time Quantitative Reverse Transcription-PCR Validation

To confirm the array gene expression data, we performed quantitative reverse transcription-PCR (qRT-PCR) for a selected set (n = 10) of genes and the representative data for three genes are shown in Table 4. Although the extent of measured values detected by the two methods varied, an overall pattern concordance between qRT-PCR and Affymetrix cDNA array experiments was observed (i.e., same trend of induction or suppression was detected by both methods for each target genes). This difference may be due to probe design or the GeneChip system hybridization conditions. For all qRT-PCR, primers specific to β-actin were used as a control to normalize each experiment. Results are presented in Table 5.

**Table 5:** Quantitative RT-PCR. Relative quantity of mRNA expression in PT, LM and NP tissues measured by quantitative real time PCR

	**Relative fold change**							
	
**Genes**	**PT1**	**LT1**	**PT2**	**LT2**	**PT3**	**LT3**	**NP1**	**NP2**
**Igfbp1**	14.4	70.0	2.0	20.0	10.0	90.0	2.0	2.0
**Sepina1a**	16.9	4.9	28.9	78.4	14.4	40.0	2.0	2.0
**Peg3**	0.4	0.2	4.9	10.0	16.0	8.1	2.0	2.0
**β-actin**	1.0	1.0	1.0	1.0	1.0	1.0	1.0	1.0

### Verification of microarray data in human pancreatic cancer cell lines

A panel of human pancreatic cancer cell lines that were reportedly to have high or low metastatic potential in immunodeficient mouse models were used to verify the data from Ela-*c-myc *model of primary and metastatic pancreatic tumors. Cell lines with high metastatic potential include PANC28, CoLo357fg, L3.6pl and low- or non-metastatic potential include PANC1 and BxPC3. We verified two genes in human cell lines, Igfbp1 and Serpina1a, these genes were highly upregulated in liver metastaic tissues compared to primary pancreatic tumors from transgenic mice. Expression patterns of both genes were consistent with the murine microarray and RT-PCR data (Fig. 2C).

## Discussion

In this study, we report the genome-wide expression profiles of primary pancreatic tumors and liver metastatic lesions from Ela-*c-myc *transgenic mice, or normal pancreas from wild-type mice. cDNA microarray analysis showed several gene clusters under various functional categories in primary or metastatic pancreatic tumors of Ela-*c-myc *transgenic mice that differ from normal pancreas of non-transgenic littermates. Notably, increased expression was observed for a large number of genes related to ribosomal biogenesis, maturation and ribosome assembly in primary or metastatic pancreatic tumors. Previous studies by others have also shown enhanced expression of genes related to ribosomal proteins, rRNA maturation and ribosome assembly, in addition to enhanced expression of many translation initiation and elongation factors in c-Myc overexpressing cells [33-35]. Thus, our model recapitulates the experimental observations and key features of c-Myc overexpressing tumors.

Genes involved in posttranscriptional regulation was a major functional category of upregulated genes in both PT and LM compared to NP samples, we observed changes in expression for splicing factors, RNA binding/pre-mRNA processing factors and spliceosome related genes, indicating that events related to RNA processing may play critical roles in pancreatic tumor development and progression induced by c-Myc. More than 50% of human genes undergo alternative splicing, and this type of RNA process has recently become an emerging topic in molecular and clinical oncology [36-38]. Our data showed upregulation of several splicing factors from the SR family such as Sfrs1, Sfrs2, Sfrs3, Sf3b in both primary and metastatic tumors compared to normal pancreas. SR proteins represent a family of essential splicing factors, which are characterized by extensively phosphorylated serine-arginine rich domains [39]. SR proteins recognize splice sites and, depending on their relative levels, these proteins can influence alternative RNA processing [40].

Other groups of genes that were upregulated are involved in DNA replication, cell proliferation and cell cycle regulation; chromosome organization and biogenesis; and signal transduction. Many genes are related to the maintenance of chromosomal structure and integrity such as minichromosome maintenance (Mcm)2, Mcm5, Mcm10, structural maintenance of chromosome (Smc)2l1, Smc4l1, Smc5l1, Rad51, Brca1 and Centromere component (Cenp-I). The entire Mcm protein family (Mcm2-7) is essential in regulating the replication of DNA. Amplification of genes in the Mcm family has been detected in various cancer cells [41]. Their upregulation may deregulate the complete and accurate DNA replication and thus result in failure to maintain the genetic integrity of affected cells. Smc family proteins are integral components of the machinery that modulates chromosome structure for mitosis [42]. Similarly, Rad51, brca1 and Cenp-I play a role in maintenance of genetic integrity [43,44]. We also noticed increased expression of some X-linked genes related to signal transduction such as Rsk4, Dusp9 and S100g, which have not been reported previously in pancreatic tumors.

Intriguingly, we observed highly upregulated expression of Igfbp1 and Serpina1 in liver metastatic tissues compared to primary pancreatic tumors and normal pancreas. Verification of Igfbp1 and Serpina1 by RT-PCR and quantitative PCR showed strong expression in liver metastatic lesions but there was a lack of expression of these genes in primary pancreatic tumors or normal pancreas. Similarly, both these genes also showed higher expression in highly metastatic human pancreatic cell lines (PANC28, CoLo357fg, L3.6pl) and lower expression levels in less-metastatic cell lines (PANC1 and BxPC3). Several studies have described the inhibitory and potentiating activities of both Serpina1 and Igfbp1 in a variety of cells [45-47]. Igfbp1 interacts with α_5_β_1 _integrin, influencing cell adhesion and migration. Jones *et al*. [48] first reported the increased migration of Chinese hamster ovary cells transfected to express human Igfbp1. Increased expression of several Igfbps has also been reported in human pancreatic cancer [28-31]. Serpins are endogenous inhibitors of serine protease activity *in vivo *[49,50] and a large number of studies support the notion that proteases play an important role in the progression of malignant tumors. Therefore, the expression of proteinase inhibitors is considered to be an anti-malignant event. Serpina1, a major inhibitor of human serine proteases in serum, is produced mainly by the liver, but also by extra-hepatic cells, including neutrophils and certain cancer cells [51,52]. However, clinical studies have shown that high circulating levels of Serpina1 directly correlate with tumor progression [53,54]. Immunohistochemical studies revealed that patients with Serpina1-positive lung adenocarcinomas had a worse prognosis than Serpina1-negative ones [55]. More interestingly, both Serpina1 and Igfbp1 have been demonstrated to play a role in human invasive and metastatic pancreatic cancer. Together these studies and our findings suggest that Igfbp1 and Serpina1 may play critical roles in tumor progression *in vivo*, and are potential candidates for therapeutic interventions.

We also compared our gene expression profiles with published data on human pancreatic cancer tissues or cell lines. Gene expression pattern of many genes such as Serpina1, Igfbp1, Wt1, CD44, MMP12, CXCR4, Muc2, Dek, Capza1, Bcra1, Birc5, S100g, Claudin-18, RAD51, Mcm2, Mcm4, Mcm7, Cyclin B2, splicing factor 3b, Nap1l1 etc. (please see Tables 1, 2, 3 and 4for references) was similarly reported in other studies and therefore provide a validation for our model.

## Conclusion

We show differential gene expression profiles under several functional categories in normal pancreas, primary pancreatic tumors and liver metastases. We identified two genes, Igfbp1 and Serpina1, which were overexpressed only in liver metastatic lesions suggesting that these genes are likely to be involved in the establishment of metastases in Ela-myc transgenic animal model. In addition, metastatic lesions appear to have low levels or absence of Wt1 gene expression while primary tumors express at least two major variants (+ exon 5 or - exon 5) Wt1 transcripts. Igfbp1 and Serpina1 may serve as clinically interesting biomarkers are likely to be useful for prognostic or therapeutic purposes in metastatic pancreatic cancer.

## Methods

### Ela-*myc *transgenic mice

We used Ela-*myc *transgenic mice with a FVB background, this strain was generated by crossbreeding of C57BL/6xSJL background Ela-*myc *[5] mice (obtained from Dr. Sandgren at the University of Wisconsin) with a FVB strain. The F1 mice were crossed together to generate F2 transgenic mice and some of the F2 mice were crossed to yield F3 mice. The F2 and F3 transgenic mice and their wild type littermates were used in this study.

### Human Pancreatic cancer cell lines

A panel of human pancreatic cell lines, PANC1, PANC-28, CoLo357, L3.6pl and BxPC3, were used to verify the microarray data. All pancreatic cell lines were cultured in RPMI 1640 supplemented with 10% fetal bovine serum, penicillin and streptomycin. Cells were harvested when they were about 80–90% confluent for RNA isolation.

### cDNA microarray

Primary pancreatic cancer tissue, its corresponding liver metastatic lesion and normal pancreatic tissues were used to prepare RNA using the RNeasy mini kit (Qiagen) per manufacturer's instructions. Assurance of quality assessment and microarray analysis were carried out by personnel in the Applied Genomics Technology Center (Center for Molecular Medicine and Genetics, Wayne State University). Briefly, biotin-labeled RNA fragments were produced from 1 μg of RNA by first synthesizing double-stranded cDNA followed by *in vitro *transcription and fragmentation reactions. A hybridization cocktail, containing the fragmented cRNA, probe array controls, bovine serum albumin, and herring sperm DNA, was prepared and hybridized at 45°C for 16 h to the High Density Mouse Genome M430-2 containing 45101 probesets (Affymetrix Inc., Santa Clara, CA). The hybridized probe array was washed, and bound biotin-labeled cRNA was detected with streptavidin-phycoerythrin conjugate. Each probe array was scanned twice (Hewlett-Packard GeneArray Scanner), the images were overlaid, and the average intensities of each probe cell were compiled. Microarray was repeated three times for each condition (LM, PT, NP).

### cDNA microarray data analysis

High density microarray image files were interpreted and quality assessed to Affymetrix standards in GCOS 1.1 as described previously [56]. Expression changes were filtered in DChip for fold change (> 4 fold) between the experiments. Hierarchical clustering was carried out using Dchip and ontological analysis of gene expression was conducted in both OntoExpress in conjunction with curated pathway analysis using the KEGG Biocarta and GeneGo systems. At least three samples from each condition were used for Affymetrix microarray analysis to select candidate genes. Candidate genes were also confirmed with semi-quantitative, quantitative RT-PCR analysis and/or western blot at least 3 times.

### Semiquantitative RT-PCR

Total RNA, isolated from the primary or metastatic lesions and normal pancreas of Ela-*c-myc *transgenic mice, was subjected to first-strand cDNA synthesis using an oligo (dT) primer and Moloney murine leukemia virus (MMLV) reverse transcriptase (Invitrogen). The primer amplified products were separated on ethidium bromide containing 1.2% agarose gels. Primers for the semiquantitative and quantitative detection of target mRNAs are presented in Table 6.

**Table 6:** List of primer. Primer sets for qRT-PCR and sqRT-PCR

**Gene name**	**Accession No**.	**Quantitative or sqRT-PCR primer sequence**
**CXCR4**		
Upstream	D87747	CATGGAACCGATCAGTGTGA (325)*
Downstream		TTTCCCAAAGTACCAGTCAGC
**MMP2**		
Upstream	NM_008610	CTGTGTTCTTCGCAGGGAAT (433)
Downstream		TGTGCAGCGATGAAGATGAT
**Snail2**		
Upstream	NM_011415	TTCCTCTGACACTTCATCCAA (474)
Downstream		TTGGAGCAGTTTTTGCACTG
**E-tcad**		
Upstream	NM_009864	CCTGCCAATCCTGATGAAAT (329)
Downstream		TCAGGGA AGGAGCTGAAAGA
**Fgf13**		
Upstream	AF020737	CATTTTCTGCCCAAACCACT (378)
Downstream		AATGCTTGGCACTCTTTTGC
**Rsk4**		
Upstream	BB402211	GTGGGTGCCAAAGTTTTGAT (351)
Downstream		CAAACCACATGGAAATCAGG
**MIF**		
Upstream	NM_010798.1	ACTACAGTAAGCTGCTGTGTGG (208)
Downstream		ATCGCTACCGGTGGATAAAC
**Mcm7**		
Upstream	NM_008568.1	ACCGCGAAGTCAGTACACAA (208)
Downstream		GATGGTCTGCTGCTCCATAA
**Ttr**		
Upstream	NM_013697.1	TGGAAGACACTTGGCATTTC (194)
Downstream		TGCTACTGCTTTGGCAAGAT
**H2Aa**		
Upstream	NM_010378.2	CCTTCATCCCTTCTGACGAT (197)
Downstream		CAGGCCTTGAATGATGAAGA
**Mrpl19**		
Upstream	NM_026490.2	TGCATCCCATGAAGAAGAGA (183)
Downstream		GACATTTGCTCGTTACAAAAGC
**Dusp9**		
Upstream	NM_029352.3	CCTGTGCTTGAGCTCTGATT (181)
Downstream		GCTCTCCAAATTGGCTGAAT
**S100g**		
Upstream	NM_009789.2	CAGCAAAATGTGTGCTGAGA (197)
Downstream		CTCCATCGCCATTCTTATCC
**Serpina1a**		
Upstream	NM_009243	GCCCTGGCAAATTACATTCT (196)
Downstream		CATTGCCTGCATAATCCATC
**Peg3**		
Upstream	NM_008817.2	ACCATTCAGGCCTCAGTTTC (205)
Downstream		TTTTCTCAAATTCGCTGACG
**Igfbp1**		
Upstream	NM_008341	CCTGCCAACGAGAACTCTAT (196)
Downstream		GGGATTTTCTTTCCACTCCA
**Saa3**		
Upstream	NM_011315.3	GCGAGCCTACTCTGACATGA (196)
Downstream		ATTGGCAAACTGGTCAGCTC
**Cldn18**		
Upstream	NM_019815.2	GCTGTACGAGCCCTGATGAT (193)
Downstream		TGTTGGCAAACACAGACACA
**Sfrs1**		
Upstream	NM_173374.3	CACTGGTGTCGTGGAGTTTG (190)
Downstream		CTTCTGCTACGGCTTCTGCT
**Sfrs2**		
Upstream	NM_013663.3	GCTTTGCTTTCGTCGAATTT (188)
Downstream		AGGACTCCTCCTGCGGTAAT
**Eif2**		
Upstream	NM_026030.2	GGAGTTGCTGAACCGAGTGT (180)
Downstream		AGGAGATGTTTGGGTTGACG
**Muc13**		
Upstream	NM_010739.1	TGCGTGATGCTACAAAGGAC (195)
Downstream		TGTCCTGGCATTTACTGCTG
**Igfbp1 (human)**		
Upstream	NM_000596.2	AAGGCACAGGAGACATCAGG (195)
Downstream		TATCTGGCAGTTGGGGTCTC
**Serpina1 (human)**		
Upstream	NM_001002235.1	TGCCTGATGAGGGGAAACTA (186)
Downstream		CCCCATTGCTGAAGACCTTA
**WT1(362–970)**		
Upstream	NC_000068	TCCAGCAGCCGGAGCAACCT (608)
Downstream		AGGGCGTGTGGCCATAGCTG
**WT1(947–1470)**		
Upstream	NC_000068	CGCCCAGCTATGGCCACACG (523)
Downstream		ATTGCAGCCTGGGTATGCAC
**WT1(1444–1943)**		
Upstream	NC_000068	TTCATGTGTGCATACCCAGG (499)
Downstream		GTAGATCCACAGTCGTGTCC

*PCR product size

### Real-Time RT-PCR

cDNA from the primary or metastatic lesions Ela-*c-myc *transgenic and normal pancreas of wild type mice were subjected to PCR amplification, a maximum of 2 μl of each cDNA sample was used per 25-μl PCR reactions. The real-time measurements were analyzed in triplicate using an automated Real Time Cycler as described previously [56]. The relative quantity in primary tumor versus normal tissue or primary tumor versus metastatic lesion was normalized to β-actin.

### Sequencing of Wilm's tumor suppressor gene (Wt1)

RT-PCR analysis using primers amplified nt947-1470 region of mouse Wt1 mRNA, which covers the first 7 exons, showed that liver metastases (but not primary pancreatic tumors) contained a lower molecular weight mRNA species. To verify the identity of the PCR products of the higher bands in primary tumor and lower band in liver metastatic lesions, we sequenced these bands using forward primer-947 after purifying them from agarose gels using Gel Extraction Kit (QIAEX II) from Qiagen.

## Competing interests

The author(s) declare that they have no competing interests.

## Authors' contributions

AT participated in the design of the study; participated in the experimental design; analysis and interpretation of data; and wrote the manuscript; AB designed primers; carried out the semi-quantitative and quantitative RT-PCR; JW isolated RNA from tissue samples and did sequencing; DJL participated in the design of the study, monitored and collected primary or metastatic tumor tissues. All authors read and approved the final manuscript.
